# Validation of early myocardial gadolinium enhancement (EGE) evaluation with "Lake Louise consensus" criteria in patients with suspected myocarditis using a single bolus of 0.1 mmol/Kg of a high relaxivity gadolinium-based contrast agent

**DOI:** 10.1186/1532-429X-17-S1-P292

**Published:** 2015-02-03

**Authors:** Nicola Galea, Iacopo Carbone, Andrea Fiorelli, Vincenzo Noce, Federica Ciolina, Marco Francone

**Affiliations:** 1 Department of Radiological Sciences, Oncology and Pathology, University of Rome "Sapienza", Rome, Italy

## Background

Early gadolinium enhancement (EGE) has been indicated as major CMR criterion for the diagnosis of myocarditis according to the Lake Louise consensus (LLC). However, the suggested threshold of 4.0 for Early enhancement ratio (EER) specifically refers to use of conventional Gd-chelates (gadopentetate dimeglumine). Our purpose was to evaluate performance of a high relaxivity contrast agent (gadobenate dimeglumine) for the detection of EGE applying the suggested cut-off.

## Methods

57 consecutive patients with a histologically-proven diagnosis of acute myocarditis and 18 controls performed a CMR study on a 1.5T unit. CMR protocol included: T2w-STIR sequences, early gadolinium enhancement (EGE) and late gadolinium enhancement (LGE) technique after a single bolus of 0.1mmol/Kg Gd-BOPTA. For each study early myocardial enhancement ratio was calculated as follows: early gadolinium enhancement ratio = enhancement myocardium/enhancement skeletal muscle. In patients with evidence for skeletal muscle involvement, an absolute myocardial enhancement of more than 45% was considered as diagnostic criterion. Receiver operating characteristic (ROC) curve analysis was applied on the overall population.

## Results

EGE was present in 35 patients with myocarditis. Applying the EGE threshold proposed by LLC (EER value>4.0) to the ROC curve, sensitivity and specificity of respectively 60.7% and 88.9% (area under curve: 0.722) were observed. These results are comparable to whose obtained by using gadopentetate dimeglumine reported in literature.

## Conclusions

Use of Gd-BOPTA provides comparable results to conventional Gd chelates regarding the application of EGE suggested thresholds for the diagnosis of acute myocarditis and it is therefore applicable in daily clinical routine.

